# Using *Candida oleophila* as a biocontriol agens to prevent foodborne *Escherichia coli* O157 EHEC infections

**DOI:** 10.1186/2193-1801-1-82

**Published:** 2012-12-28

**Authors:** Yujian Wang, An Wei, Hongyu Li

**Affiliations:** 1 Key Laboratory of Environmental and Applied Microbiology, Chengdu Institute of Biology, Chinese Academy of Sciences, Chengdu, 610041 People’s Republic of China; 2 Department of chemical engineering, Boston University, Boston, MA 02215 USA; 3 Institute of Microbiology, School of Life Sciences, Lanzhou University, Lanzhou, 730000 People’s Republic of China

**Keywords:** *Escherichia coli* O157:H7, *Candida oleophila*, Apple wounds, Response

## Abstract

*Escherichia coli* O157:H7 (EHEC O157) is a serious pathogen causing haemorrhagic colitis. In this study, inactivation kinetics of inoculated EHEC O157 and response of EHEC O157 in apple wounds by different concentrations of *C. oleophila* was investigated. The results presented in this study indicated that EHEC O157 could survive and grow in wounded ‘Fuji’ apples and extensively proliferate. Water as a decontaminant was ineffective in reducing EHEC O157 in wounded apples. *C. oleophila* could be a viable method of controlling wound contaminated by this pathogen at room temperature.

## Introduction

Foodborne bacterial infections can affect high numbers of people with large-scale outbreaks occurring. There have been a number of outbreaks due to *Escherichia coli* O157:H7 (EHEC O157). EHEC O157 was first recognized as a human pathogen in 1982 during the investigation of two outbreaks of bloody diarrhoea in Oregon and Michigan (USA) (Riley et al. 1983). Outbreaks of the infections have now been reported from United States and Canada (Bell et al. 1994), Asia (Michino et al. 1998), Australia (Desmarchelier 1996) and Europe (Williams et al. 2000).

One of the biggest problems with controlling EHEC O157 outbreaks is the low infectious dose required for infection to occur (Strachan et al. 2001; Teunis et al. 2004). This means that even slight contamination of surfaces or work areas may cause serious infection. Many vegetable and fruit products such as apple, raspberries and strawberries can be contaminated. Injuries to apples through stem punctures, bird pecks or wounds created when they drop and by other various types of physical abuse may permit entry of pathogens such as EHEC O157 from animal feces, contaminated water, dust or soil (Riordan et al. 2000). Dingman (2000) reported the ability of *E. coli* O157:H7 to survive and grow in the areas of injury on an apple, and thus, there exists a food safety risk associated with those apples to be consumed raw or destined for the production of unpasteurized apple cider. Recent outbreaks of foodborne illness associated with consumption of fruit juices, in particular apple cider, has led to published regulations by the U.S. Food and Drug Administration (FDA) proposing mandatory adoption of hazard analysis critical control point (HACCP) programs (FDA Food and Drug Administration 1998; Annous et al. 2001). In this way, EHEC O157 presents a serious public health risk from cross-contamination from surfaces to food produce.

Yeasts have been studied for more than two decades as biological control agents against postharvest diseases caused by fungal pathogens of fruit. Wounded fruit, in particular, needs protection because wounds are primary sites of infection by several postharvest fungal pathogens. *Cryptococcus laurentii*, *Pichia guilliermondi*, *Kloeckera apiculata*, *Sporobolomyces roseus*, *Candida sake* and *Candida oleophila* (Usall et al. 2000,2001; Abadias et al. 2001; Gamagae and Sivakumar 2004; Lassois et al. 2008) have shown biocontrol effectiveness against postharvest diseases caused by fungal pathogens of apple. Recently, inhibition of *E. coli* and other enterobacteria by *Pichia anomala* and *Hansenula anomala* yeast in storage systems has been shown by M. Olstorpe and colleagues (Olstorpe et al., 2010,2012). There is few scientific literature about application of *C. oleophila* yeast as antagonist for controlling of EHEC O157 in vegetable or fruit products.

In this study, inactivation kinetics of inoculated EHEC O157 in apple wounds using *C. oleophila* was studied. The main objective of this research is to investigate the feasibility and effectiveness of *C. oleophila* for reducing population of EHEC O157.

## Materials and methods

### Apples

Unwaxed ‘Fuji’ apples were purchased from a local grocery store. Apples were of uniform size (230±10 g) and were free of visible defects such as bruises, cuts, or abrasions. The apples were stored at 2°C and used in experiments within 7 days. Before use, the apples were washed with 2% sodium hypochlorite for 2 min, rinsed with deionized water, and air-dried before wounding.

### Microorganisms

Clinically isolated EHEC O157 was kindly provided by Third Military Medical University (Chongqing, China). Cultures propagated at 30°C for 20 h in 10 ml of tryptic soy broth (TSB), were harvested and washed three times by centrifugation at 2000 ×g for 10 min at 4°C in 0.9% (w/v) sodium chloride solution to yield an approximate population of 1×10^7^ CFU/ml.

Yeast *C. oleophila* strain was isolated by Bioengineering College of Lanzhou jiaotong University, Lanzhou, and stored at −80°C in glycerol solution (50%). The cultures were maintained on nutrient-yeast extract-dextrose Agar (NYDA) slants (8 g of nutrient broth, 5 g of yeast extract, 10 g of dextrose, 20 g of agar and 1000 ml of water) at 4°C. Nutrient broth was purchased from Qingdao Hope Biotechnology Co., Ltd. China. *C. oleophila* was grown at 24°C for 48 h in 500 ml shake flask cultures in nutrient yeast broth (NYDB). Then the cells were harvested at 5000×g for 10 min at 4°C and resuspended in deionized water (1×10^8^ CFU/ml). Desired concentrations were adjusted with the aid of a hemacytometer.

### Apple streatments

Fruits were wounded at five sites about midway between the stem and the equator with a metal bar which was pressed against the skin until it ruptured, making triangular wounds of about 4 mm each side. Each wound was inoculated with 20 μl *C. oleophila* suspension and 20 μl EHEC O157 suspension (10^7^ CFU/ml). The concentrations of inoculated *C. oleophila* were 10^8^ CFU/ml (K8), 10^7^ CFU/ml (K7), 10^6^ CFU/ml (K6), 10^5^ CFU/ml (K5), 0 CFU/ml (K0), just inoculated with EHEC O157) respectively. Then the apples were placed in perforated plastic trays and stored at 25°C. Following treatment, the apples were rinsed by dipping in sterile water at 25°C for 30 s and left to dry.

### Sampling and microbial analysis

The concentrations of yeast and EHEC O157 population were monitored every day. On each sampling day, three apples were removed from each tray and the wounds were taken from each fruit using a sterile cork borer of 13 mm in diameter. Then the wounds were placed in a sterile plastic bag with 90 ml of 0.1% peptone (w/v) solution and blended. The resulting suspension was serially diluted in 0.1% peptone and 100 μl was applied to Petri plates in triplicate. *Candida oleophila* was isolated on malt extract agar supplemented with streptomycin sulphate (100 μg ml^-1^). Yeast colonies were counted after three days at 24°C. EHEC O157 was isolated on tryptic soy agar (TSB) incubated at 37°C for one day to determine colonies. An analysis of variance of the log_10_ transformed data was carried out for each sampling day.

### Statistical analysis

All experiments were replicated three times. The data presented are the means of triplicate experiments. Significant difference was determined at 5% level of significance (P<0.05) by Student’s *t*-test.

## Results

**Figure 1:**
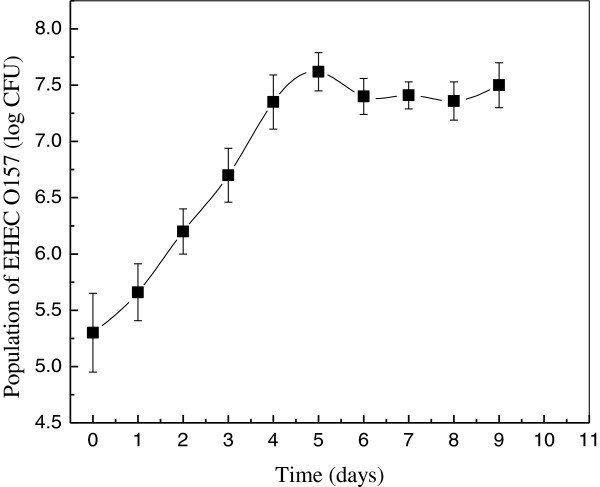
**Concentration dynamics of EHEC O157 during storage at 25°C without*C. oleophila*.
**

Concentration dynamics of EHEC O157 in apple wounds without inoculating with *C. oleophila* was investigated. The initial population of total EHEC O157 was about 5.5 log CFU in an apple wound. Population dynamics of EHEC O157 in wounded apple during storage period in this study are shown in Figure 1. The EHEC O157 population decreased during the first 24 h at 25°C. Subsequently, it increased progressively to reach maximum population of 7.6 log CFU/wound after about 5d of storage. After that the population remained stable. Water treatment was carried out at day 6. However, it caused little change in pathogens count with a reduction of about 0.4 log CFU/wound of EHEC O157

**Figure 2:**
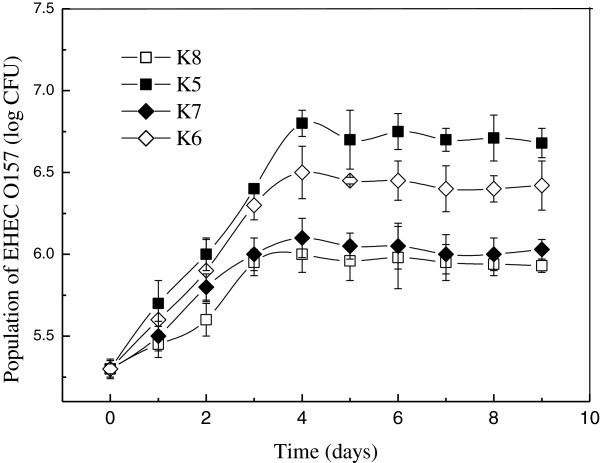
**Concentration dynamics of EHEC O157 during storage at 25°C with*C. oleophila*.
**

Concentration dynamics of EHEC O157 in wounded apples during storage at 25°C after treatments by different concentration of *C. oleophila* is shown in Figure 2. Inoculum of *C. oleophila* showed a significant effect on development of EHEC O157. The higher concentration of *C .oleophila* inoculated, the smaller increase of EHEC O157 was. Compared with K8 (*C. oleophila*, 10^8^ CFU/ml), K5 (*C. oleophila*, 10^5^ CFU/ml), as applied in this study, did not result in effective reduction of EHEC O157 populations in the first 12 h, but was able to significantly (P < 0.05) impede growth of the pathogen at the following time. The maximum populations of EHEC O157 inoculated with K5, K6, K7 and K8 were 6.8, 6.5, 6.1, 6.0 CFU/wound after 4 days of storage at 25°C, respectively. So K7 (*C. oleophila*, 10^7^ CFU/ml) could be most appropriate for prevention growth of EHEC O157 at room temperature.

**Figure 3:**
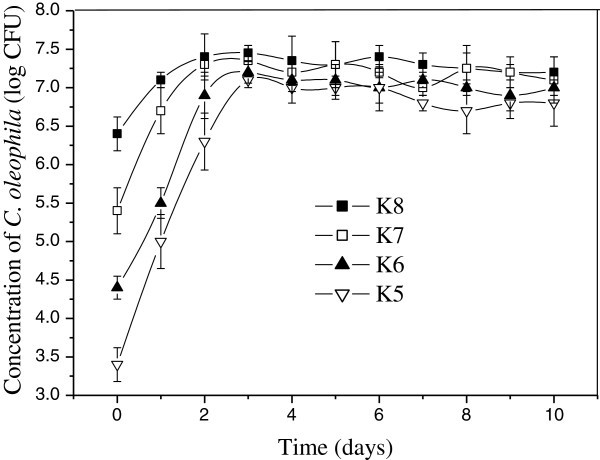
**Concentration dynamics of*C. oleophila*during storage at 25°C with EHEC O157.**

The *C. oleophila* populations inoculated with EHEC O157 increased evidently to maximum populations during the first 2 days following its application to wounded apples with little changes thereafter. The result was presented in Figure 3. Mean maximum populations of *C. oleophila* recovered from apples did not differ significantly each other regardless of initial inoculum concentrations.

## Discussion

Water treatment didn’t cause a significant change in pathogens count with a reduction of about 0.4 log CFU/wound of EHEC O157. This was less than that reported by similar research evaluating the rinsing effect on apple surfaces previously (Ruiz-Cruz et al. 2007; Wright et al. [Bibr CR21_70][Bibr CR22_70]). Attachment would be stronger in wounds due to the fibrous structure and porous nature of the apple tissue as compared with the smooth. These studies demonstrated that EHEC O157 could grow at room temperature and the use of water is insufficient to eliminate the pathogen in wounded ‘Fuji’ apples. Therefore the use of effective antagonist is necessary to prevent growth of EHEC O157.

The stoppage in growth of biocontrol agents in fresh wounds after the rapid initial increase is likely to be due to the population exhausting certain factors and reaching the carrying capacity of the wound. The result showed that the *C. oleophila* could grow well in wounded apples. A 4-log increase of *C. oleophila* (K5) was attained after 3 days of storage at 25°C. Similarly, previous studies showed that antagonist populations could grow exponentially in Red Delicious and stabilize after their application to wounds regardless of the population level or the addition of supplemental nutrients (Mercier and Wilson 1995). Furthermore, no other organisms were isolated in significant numbers during the course of this experiment. Athough apple wounds are probably colonized by other natural bacteria (Mercier and Wilson 1994), it is unlikely that they could be important enough to interfere with growth of *C. oleophila.*

From discussion above it can be concluded that EHEC O157 could survive and grow in wounded ‘Fuji’ apples and can extensively proliferate at room temperature. The use of water as a decontaminant was ineffective in reducing EHEC O157 in wounded apples. *C. oleophila* effectively reduced the development of EHEC O157, at the same time the concentrations of *C. oleophila* also affect the growth of the pathogen significantly. However, there were no significant differences in *C. oleophila* populations recovered from wounded apples. Further studies need to be conducted to address the issue of effect factor and interaction mechanism between the yeast and bacteria.
